# Diphtheritis or Diphtheritic Sorethroat

**Published:** 1861-02

**Authors:** T. A. Means

**Affiliations:** Memphis, Tenn.


ATLANTA

Medical and Surgical Journal.

Vol. VI.] FEBRUARY, 1861. [No. 6.

### ORIGINAL COMMUNICATIONS.

### ARTICLE I.

*Diphtheritis or Diphtheritic Sorethroat.* By T. A. Means, M. D., Memphis, Tenn.

Among the various inflammatory disorders that affect the throat and air passages, there is none, perhaps, which at times, has committed such ravages, and been less amenable to medicine than that popularly known as Diphtheria or pseudo-inflammation ot the fauces.

The numerous titles given to this affection have been introduced more as a novelty in nomenclature than a refinement of time; I shall therefore omit a classification of them, and content myself with the notice of one or two of the most important distinctions, only.

Secondary Croup of Dr. Stokes, Diphtherite, Diphtheritic sorethroat, and pellicular inflammation of M. Bretonneau, have been given to designate a particular disease which has for its characteristic feature, a tendency to the formation of a false membrane, whether affecting the epithelium, or dermoid tissue. Under the head of which, the latter author has embraced not only the acute and gangrenous varieties of laryngitis, but also inflammation of the trachea, contending moreover, that this peculiar disease is identical with croup, originating from the same cause and requiring the same treatment. The titles mentioned are by no means synonymous nor the symptoms of each identical. Yet, there are traits sufficient in common, to justify a notice of these conjointly.

The lesions implied by the term Diphtheritis are by no means of modern origin. Full and accurate details have been furnished by various authors, anterior to the present century, yet classed under the head of malignant angina as a sequence, rather than a disease *sui generis*, whilst others of our own day and time contend for and attempt to establish its identity with erysipelas, scarlatina, &c., whilst a third suppose it to be a specific poison taken into the system and seen at the throat.

These with other complex theories respecting its orgin, pathology, anatomical characteristics and treatment, tend more to confuse than enlighten the patient, student or ready observer. The term Diphtheritis would be more appropriately applied after the inflammatory symptoms have subsided, and the exudation assumed a membranous form. Yet by popular acceptation the title embraces every grade and feature from outset to termination. It is not my purpose here to answer the right of nomenclature, but simply to record what observation has taught me in the treatment of 27 cases which have fallen under my care since the appearance of the disease, in March or April last.

The different forms, or stages of this affection, as it has presented itself to me, may be classed under three heads, presenting the following conditions and anatomical peculiarities :

1st.—A diffused membranous concretion or deposit of colorless fibrin, distributed in patches, shading into one another, easely detached and with no congestion.

2d.—A thickening of the exudation, more extended, of a darker hue, and accompanied with offensive breath.

3d.—Increased enlargement of the tonsils, further extension of the deposit, tense engorgement, and a free putrescent discharge from the nares.

The destinctive feature of the disease, however, is the great readiness with which the exudation is detached and reproduced, each succeeding layer increasing in color and consistence until it becomes that of a tough elastic coating difficult of displacement, and which, if allowed to go unchecked, extends by continuity to the parts adjacent, soft palate, and pharynx reaching eventually the larynx, producing croupy symptoms with addition of an inability to swallow. The breathing becomes deep and sonorous; the voice is reduced to a sort of subdued whisper; paroxysms of suffocation supervene, then death.

This is the ordinary condition of things in most cases after the formation of the membrane. Yet there are others from which patients recover, after the concretion has been detached, the disease becoming less persistant, and but little disposed to reach the air passages, in which case, there is very little reason to fear a fatal termination.

The symptoms indicating its approach varied but slightly in 19 of the 27 cases above alluded to, whilst 7 of the number, presented peculiarities it would not be amiss to notice. I have therefore chosen them, as they best show the characteristic features of the most important distinctions mentioned on pages 3 and 4, reserving the remainder to be noticed under the head of diphtheritic sorethroat.

CASE I.—I was called, 8 o'clock, A. M., 12th April last, to see a negro child of 10 months, fleshy and of active habits, had been ill for four days with what was supposed to be Aphtha. The following conditions were observed: skin moist; temperature uniform; pulse 120; breathing croupy and difficult; bowels torpid. Owing to the tender age of my patient, the great prostration, and external enlargement in the region of the neck, I was unable to make a satisfactory examination, and was forced, therefore, to rely mainly upon the very imperfect history of the case given me by the mother. To satisfy myself in making out a more satisfactory diagnosis—this being my first case—I pushed my investigation further. Yet with great inconvenience—respecting the fauces I found the tonsils swollen, indurated, and covered their whole extent with a greyish colored exudation, soft, smooth and adherent, the uvula thickned, pyriform and directed back towards the pharynx and resting upon the lower border of the tonsils. At the base of the tongue, forward of the velum paliti, were several small, elevated, opake patches or concretions, seemingly upon, rather than out of the mucous tissue, which even at this period were easily detached, and when made to disappear, left behind no pit or excavation. This simple circumstance, lead me to suspect the existence of diphthritis; I therefore instituted the following treatment, relying cheifly upon remedies advised in our books: ordered first a simple emetic; mild but continued counter-irritation over the upper part of the chest; the throat mopped with a solution of nitrate of silver, 60 grains to the ounce of water; mild chloride of mercury and tartarized autimony each two grians; and patient confined to bed under light covering.—Returning at 6. P. M. found medicine had acted imperfectly; pulse, at the wrist, 100 small and feeble; breathing 20 and difficult; cough less croupy—prescribed

℞. Quinine Sulphatis

Potassa chloratis aa ʒ ss.

Aquæ, ℥ iv.

teaspoonful every second hour, and after each portion the following wash to be applied frequently through the night:

℞. Potassa Nitras ʒ ss.

Pulv. Acacia ʒ ii.

Aqua Rosæ f ℥ x.

a nutrient clyster of beef broth and arrowroot to be given twice a day.

13th, 9 o'clock A. M., no perceptable effect of the medicine; noticed change for the worst; inspiration difficult and fatiguing; breath exceedingly offensive; great depression; no discharge from the nose. A second examination of the throat, revealed an altered condition of the mucous membrane, in appearance, extent and consistency, embracing the whole posterior portion of the pharynx dark brown—approaching black, dry, smooth and exceedingly adherent. Immediately thereafter, the patient expired as if by suffocation.

Case 11. A child, aged 18 months, whom I had treated a fortnight before for dysenteric diarrhœa.

The first symptoms of its approach was observed by its mother, the night previous to my visit, and described to me as a simple sorethroat, pain and stiffness in the muscles of the neck, difficult swallowing, lassitude and slight fever.—Upon inspection I found the tonsils inflamed and slightly congested, having upon their surface, small milk-like patches, as in the preceding case, somewhat elevated and irregular, which, on being broken up left the mucus membrane beneath entire; yet of a deeper red than the parts adjacent.

The paroted and submaxilary glands were slightly enlarged, doughy and tender to the touch, the face wore an anxious expression, the eyes dull and watery.

Having the case under care, and at this early period, I anxiously sought the appliances at hand, confidently hoping to cut short the disease in its incipiency. Calling for a tablespoonful chloride of sodium, with three or four of water, I made a solution and injected it with a small glass syringe back against the fauces, two or three times, successively, applied a small flannel sack filled with hot salt to the throat—from ear to ear, confined patient to bed, and ordered weak broth, chicken water and warm sage tea to be allowed whenever called for.

At my morning's visit, 9 o'clock, I was pleased to find the strength of my patient improved, the prostration of the evening previous, having given place to restlessness and a desire for food. The condition of the soft parts within the fauces excited my fears, leading me to suspect a fatal issue. There was a soft consistent exudation enveloping both tonsils, velum palati, and lower half of the tongue. At every inspiration, and expiration, there seemed to be a preternaturally augmented motion of the larynx, and when the breath was drawn in, the air entered and was returned impededly, as if forced through a narrow fissure; the voice was husky and labored, the cry inaudible and converted into a simple play of features. In this condition I prescribed

℞. Tinct opii, gutt. xvi.

Vin. Ipecae, gutt. xii.

Spirit, Ætheris, Nitrici, f ℥ i.

a teaspoonful every half hour until free diaphoresis was produced, the solution—chloride of sodium—to be injected and repeated as necessity required, and the salt sack replaced warm as before.

6 o'clock P. M., a slight alteration in the general symptoms, the local remaining unchanged; requested same treatment to be kept up until my next visit.

Having to leave the city, did not see my patient until the following evening and fourth day, when a better condition of things was observed than at any period since the attack. The medicine had acted well, leaving the system free from that unpleasant depression so prominent in the outset. Deglutition seemed yet annoying, whilst the pain and swelling at the angle of the jaw, had, to a great extent, subsided.—The concretion had become soft, here and there detached in patches, especially that portion covering the tonsils, and while attempting to introduce my finger for the purpose of displacing it, an effort at coughing was made which brought away two or three fragments the size of a two bit (25cts,) piece, irregular, and resembling shriveled epidermis. Breathing became instantly free, deglutition performed with ease, and the depression seemed lessened with a manifest moderation of all the more prominent symptoms.

I ordered, immediately, a strong solution of alum and port wine to be injected upon the parts denuded. At my morning's visit, 8 o'clock, I found a second deposit had been made, but fortunately, less dense and adherent. This was broken up, as also a third, each less consistent, leaving the sub-mucous surface red, with here and there patches of small granular papilæ—left patient easy and comfortable.

6 o'clock P. M., had slept five hours; pulse 96 and full; respiration 16, free and easy, ordered quinine in half grain doses every hour, in one ounce weak brandy; beef broth *ad libitum.*

10th and sixth day, improvement progressive—discharged

TREATMENT.—Recapitulation—solution chloride of sodium by injection to fauces; diaphoretics; quinine; brandy; port wine; salt sack to throat, gentle cathartics.

CASE III.—A lad aged 8 years, son of Mrs. H., residing four miles from the city, to whom I was called sometime in August last. But little attention was paid to him, suppos ing his case to be one of simple angina. The following history, dating back to the onset of the disease, was received: Ordinary sorethroat was the symptom, accompanied with great muscular prostration, listlessness, loss of appetite and a general disposition to stroll about the premises, without having the physical strength to meet his desires.

On the first day of attack, the fauces appeared greatly inflamed, the soft parts in the region of the neck were not swollen but tender. Free cauterization was made with caustic in stick and forty grains to the ounce, two or three times daily, with chlorate Potassa every three hours. Up to third day of attack his strength seemed to attend him, when, on the morning of the same day, he became prostrate and helpless. The glands at the angle of the jaw began to swell; swallowing difficult; the face puffed and expression anxious.

On the fourth day, morning, thick grumous patches, white and irregularly distributed, were seen upon the latteral walls of the fauces, tonsils and palatine arch which in ten hours spread and became a thick membranous coating, lining the whole interior of the fauces, the pulse ranging from 90 to 103. This skin was constantly moist, bowels free and a frightful difficulty in breathing. The cautery was again applied; essence of beef, with arrowroot jelley were administered as long as received by the mouth, afterwards by enema. At 12 o'clock, same evening, I saw patient for the first time, with the following appearance and symptoms: prostration complete; the tongue livid at the point and on its edge, and coated throughout with a dry leathery deposit; deglutition was impossible; respiration rapid. Examining his hands, shoulders and mouth, I discovered a number of bullæ or blebs, filled with a thick gummy secretion, resembling in every respect that seen on the mucous membrane of the fauces. Instead of producing a thick brownish crust or pellicle, as the former, they became flaccid and dry by absorption, leaving the epidemis entire. One or two were broken when all the characteristic features of the true diphtheritic deposit were exhibited—even in color. Upon these I made a free cauterization with caustic in stick, but with little benefit.—Seeing the strength of my patient fast giving way, and with but slight hope that remedies would reach the necessities of his case, I sought to remove the membrane, and thereby, if possible, lessen the inconveniencies attending respiration, but was unsuccessful. Nutrient clysters, the free use of wine-whey, with occasional astringent applications to the throat, a teat or pledged of soft linen saturated with a solution of gum and sugar, was kept in the mouth to allay dryness, and facilitate deglution, and the movement of the tongue which was exceedingly dry, smooth, and turned up at the tip. I requested the suspension of the caustic and urged the necessity of the continued use of stimulants, diaphoretics, nutriment by injection, and the constant application of a warm poultice to the throat.

I learned on the following morning that a portion of the membrane had been cast off, and the general condition of the patient better. This was of short duration, however; a relapse supervened and hastened death fifth day.

CASE IV.—I was called 16th August, at the instance of Dr. Willett, to see a family of sick negroes, twenty-five miles below on the river, reported sick with a singular disease.—

Found ten down suffering from sorethroat and dysphagia, two having all the symptoms of diphtheritis. The eldest—a boy of nine years—presenting the aspect of one with impending suffocation; expression anxious and alarmed; skin soft and cool; pulse 92 and feeble; respiration 26. The mucous membrane of fauces—especially its lateral walls—exhibited a tense unyielding coat, buffy and extending as far back as could be seen; breath exceedingly offensive; profuse saliva, and an acrid discharge copious and scalding, from the nares. There was no great enlargement in the region of the neck, nor was there much heat. The expression of the eyes were dull and unsteady; the voice husky and labored, slight cough, croupy and short, with no expectoration; bowels torped; frequent desire to stool. Ordered mercury in grain doses every hour until five were taken.

7 o'clock A. M., no improvement; general condition same as before; expression of alarm less observable; great pain in swallowing; becomes rapidly exhausted; pulse 84; respiration 21. Gave Quinine in two grain doses, every two hours; brandy and beef tea, *ad libitum.*

12 o'clock, M., general aspect worse; skin moist; tongue red, dry and turned up at the point; answers questions by signs; the exudation extends further back than when seen in the morning, compact and of a dirty brown. Ordered cauchandal plaster to throat, chlorate of potassa in teaspoonful doses every three hours intermitting with a gargle of alum and port wine—tepid pediluvium, and patient confined to bed.

10 o'clock P. M., aspect worse; is exceedingly restless; urine albuminous; tongue sphacelated dry and tender; eyes upturned and partially closed; pulse at the rist thready and almost lost; extremities cool, with approaching dissolution. Wishing to detach a portion of the membrane for inspection, I forcibly introduced a probang charged with a strong solution nitrate of silver, far down into the larynx, but succeeded only in securing a mere fragment.

2 o'clock P. M., patient sprung from his couch; fell back and expired.*

CASE V.—Boy Ned, similarly affected, yet with less complete prostration; great difficulty in swallowing; could not speak above a whisper; coughed but little; bowels torpid, had taken five grains of mercury before my arrival; urine scant and, as in the preceding case, albuminous—skin sleek, moist and warm; gave acetic acid, muriate of ammonia; honey and water as a gargle every twenty minutes, salt sack to throat.

10 o'clock A. M., slight improvement; pulse 80, full and soft; eyes puffed; thirst moderate; deglutition painful, but performed with less effort; breath offensive. Ordered vegetables, boiled milk, teas, &c., abstinance from all animal food and a free use of acid drink. Kept patient in hot pediluvium twenty minutes, and legs wrapped in warm flannel.

2 o'clock P. M., improvement progressive, but slight fever; returning appetite, and could swallow with less difficulty—discharged. The remaining five, all had sorethroats with small ulcerated patches scattered here and there, between which could be seen the mucous membrane. I ordered in the outset emetics, mercurial cathartics, poultice to the throat, gargle of alum, quinine and brandy; pediluvia at bed time; patients kept in doors; all daily food to be refused, and the simplest diet enjoined. One week after, I met Mr.

---

*It is proper to remark, that the solid nitrate of silver had been freely applied to the throat once or twice, by a neighboring physician previous to my arrival.

B. on the street, and was informed that all were up and convalescing. One or two more cases had occurred of ordinary sorethroat and the silver applied—both of whom died.

Two or three weeks after, I was again called. Found down with same affection, one, aged 2 years, with true diphtheritis, yet of a different type from those reported on the preceding page, *i. e.* the absence of fetor and enlargement in the region of the neck; had taken five grains of mercury through the day, producing free and copious stools; pulse normal; surface cool; prostration excessive with considerable dysphagia and utter loathing of food; breathing low and sterterous.

Finding patient rapidly sinking, did not prescribe; died within thirty minutes from first sight—post mortem refused.

Looking into the throat, I found abudant faucial concretions extending over both tonsils, down the larynx, of great thickness and exceedingly adherent. Two had died previous to my visit, one described as being malignant of four days standing, was first attacked with ordinary sorethroat. Nitrate of silver was applied, followed by gargles of alum and cayanne pepper, but with little benefit. On the third day the symptoms became aggrivated, the tongue coated and black; died from suffocation. Another, a child of 8 months; no sorethroat existence of membrane; breathing short and rapid; unusual prostration. Upon the lips, corner of the eye, on the wrist, ankles and cheeks, small isolated blebs of serum appeared, which soon became confluent—forming a concrete mass, and when ruptured presented a pseudo-membranous couch resembling that seen upon throat in diphtheritis proper, carthartics, diaphoretics and astringent gargles had been given, but with no relief.

Diphtheritis essentially a disease of debility, and whether benign or serious, is seldom unaccompanied with sorethroat. This pain in the throat is also met with in various exanthemeta, such as variola, measles, scarlatina, &c., accompanied by minute pustules upon the mucous membrane of the fauces, which might be readily taken for the disease in question.

*Anatomical appearance.*—From the first day of infection, the palatine arch is of a pale red, analogous to the skin, yet somewhat deeper, the tonsils are enlarged and of a purplish hue. After the disease has progressed for some days, minute yellowish concretions appear upon one tonsil, sometimes upon both, yet ordinarily they are affected simultaneously. Examining them more closely after being detached with an ordinary probang, they will appear shredy, or fibrinous bearing a close resemblance to the first or formative stage of the diphtheritic membrane. When seen upon the fauces in ordinary scarlatina, or epidemic angina, they are pulpy in appearance, resembling the gill of an oyster, or simulating very closely, the surface of a bad ulcer after being cauterized.

As the disease progresses, in the same ratio does its intensity. Anginose symptoms increase so as to interfere with respiration, and deglutition—especially the latter. Sometimes so great is the dyspagia, that fluids taken into the mouth return by the nose, as in scarlatina, the voice becomes dry and nasal; the ganglia in the region of the neck begin to swell, especially the parotid and submaxillary, usually the former.

At this period, direct application should be avoided, as the shredy deposit will retrocede with the disappearance of the eruption at the base of the tongue, leaving the surface red and moist. This condition of the parts left exposed will lead one to believe the disease at an end; but upon inspection, the tongue and fauces will be found smooth and sensitive, which excess remains persistent, and with it increases other symptoms, such as thirst, depression, &c.

This is the condition in ordinary cases, before medication. There are others certainly more violent, yet seemingly less severe. One form, at least, I must not omit to mention. I allude to that in which the patient is attacked with moderate severity, slight delirum, or wandering at night, tossing of the arms, with general restlessness; the pulse quick, and moderate soreness of the throat.

Should the disease pass to the sixth or eighth day, with no more aggravated symptoms than those enumerated, recovery would seem certain; but suddenly considerable engorgement is noticed at the angle of the lower jaw, affecting not only both sides, but the neck, and sometimes the cheeks and eyes.

At this period, as in cases III and V, a sanious discharge, somewhat consistent, yellow, fetid and abundant, flows from the nose; the tonsils become greatly enlarged, buffy, and pointed; the breath is exceedingly offensive; the pulse rapidly becomes soft, frequent, and yielding; the unsteady aspect of the face reappears; the skin is rough and cold; coma succeeds, and the patient succumbs, as if by suffocation, or lingers for hours in frightful torture.

These phenomena are almost always complicated with scarlatina—especially malignant—before the croupal affection has had time to reach the larynx, and it is this close resemblance that renders a ready and correct differential diagnosis exceedingly difficult. The most prominent difference being, it appears to me, in the extent rather than the anatomical appearance; the one, scarlatina, seems not only to affect the skin, but the glandular system, confining itself with peculiar activity to the throat, whilst the false concretions are limited to the nasal passages, ears and fauces.—Malignant diphtheritis, presents a striking similarity to some of the phenomina just cited, so much so, as to tempt the belief that it is not scarlatina, but the last dreaded affection. Two of the cases mentioned on the preceding pages certainly presented some of the leading features of scarlatina maligna *i. e.* invasion of the larynx, suppuration of the glands &c. &c. Cases have been recorded, where death resulted from cynanche, following scarlatina after eruption had disappeared, leaving behind no trace of the disease, save the tuberlar false membrane, upon or cast from the trachia.

I can scarce believe that the phenomena mentioned are any other than those of scarlatina anginosa, with diphtheritis as a complication, for in both we find unusual palor, cool skin, thready pulse, fetid breath, both by the mouth and nose, with partial or general dissolution of the mucous tisue.

Causes—There are certain peculiar conditions in an epidemic atmosphere where measels, scarlatina, erysipelas, small pox, &c. have prevailed, that favors the production of a peculiar crasis of the blood, which may be considered the germ or source from which springs the diphtheritic manifestation, in the same manner, as excoriations around a wound left exposed to the gyneotic virus, produces erysipelas.—Leave one surrounded by this atmospheric poison—especially if he be at all delicate—and we find the throat sore, with a diffused red efflorescence together with other symptoms—essentially those of scarlatina, such as adynamia, putrescence, dysphagia and albuminous urine—this latter not always present. There is one thing it is proper to observe, that soreness of the throat with great intumescence, fœtor, and physical depression, with erythematous eruption, appearing at the close of epidemic scarlatina, will almost surely lead to a fatal issue, whereas, if the eruption appears at the outset and continues until the fifth or sixth day, a fatal termination need not be feared, and we should resist, as far as possible, the temptation to administer drastic purges, apply caustics, and dose the patient with the view of urging on the elimination of this morbid product; it would be a species of heroism that we should deprecate. It results more frequently from reckless management, or over zeal in eliciting effect, or perhaps, through fear lest such an innovation upon custom would hasten on the already impending crisis. Thus, from the foregoing, it is reasonable to suppose that the disease in question is but a *diphtheritic complication* of scarlatina.

A slight digression may be pardoned just here, to note three forms of disease that have prevailed in this city and suburbs, since the opening of the present year, all of which were febrile and of epidemic re-currence; measels made its appearance toward the close of March, in some localities severe, but not generally fatal—in others slight, and of short duration. Immediately upon this cause diphtheritis, the first case occuring 15th April, and continuing with signal fatality through the Spring and Summer months, up to September, when dengue or "eruption break bone" made its appearance, rapidly affecting two-thirds of the entire population, irrespective of sex, or conditions—children being usually exempt, yet strange to say, not one death in one hundred occurred.—Now in the first and last named, that peculiar erythematous eruption, and desquamation was observed, as in scarlatina, yet at a more advanced period, while in the two first, the throat and air passages, were in all cases affected. The symptoms most prominent in "eruption break bone"—and certainly the one most complained of is arthritic pains, or as generally described by patients, muscular soreness.

In the acute period of scarlatina—before any ganglionic enlargement is observed—we fine precisely the same state of things. In diphtheritis we observe enlargement of the glands, the rapid production of a viscid phlegm, great muscular prostration, not soreness, and not unfrequently, as in cases II and III, a patulous eruption, occupying the extremities. I cannot go into further detail, and have only alluded to these, to show that there exists in the three affections, features so closely allied to scarlatina, as to warrant a belief of their identity. Yet, why there occurs a fever with accompanying arthritic pains, red exanthem, deposition of plastic lymph, and nervous or muscular prostration, remain yet to be satisfactorily explained, and it must require a long series of observations, during several epidemics, with the most careful analysis of all attendant symptoms, the progress and termination, before we could pronounce with certainty, that they are identical. I hope these hints may provoke a spirit of research, from able heads, and thus bring to light the causes of a disease yet obscure.

*Morbid Anatomy*—In the study of the morbific process effecting the cell growth between the outer surface of the mucous membrane and epithelium, we have but little foundation for believing it the germ or source from which springs the earlist exhibition of the diphtheritic exudation. Take, for example, simple catarrh, and the most prominent phenomina observed are red efflorescence, with alternate humidity and dryness of the epithelium, producing that abstructed sensation felt in the upper or anterior nares. This, after a lapse of time, gives place to an agreeable moisture produced, doubtless, by a sort of liquid exudation through the capillaries, to the mucous surface. This reproductive process continues, each succeeding, being more and more albuminous, yet wholly wanting in fibril texture, or structural eliment, whereas, in the diphtheritic exudation, the effused liquid plasma, almost immediately after production, forms a thin glazing, either partial, or entire, over the mucous surface of the fauces and of variable thickness, in the structure or body of which, may be seen—with the aid of a lense—degenerated epithelial cells, interlaced with minute fibers, giving to the preparation its toughness and density. I have examined but one specimen taken directly from the fauces, and that was detached so irregularly I could not, with satisfaction, give its nature, yet it appeared as a fibrinous reticulum, exhibiting the nuclii of imperfect cells, imbedded in a sort of granular infiltration springing up out of the mucous tissue, and having for its base a dense, muddy looking substance, characterised as the diphtheritic membrane. It has been my purpose to extend these investigations further, by microscropic examination of all forms of deposit in throat affections—form the ordinary phlegm expectorated in simple catarrh, to the pseudo-membranous exudation moulded upon the trachea in croup.

Flattered by the exhibitions already obtained, I am induced to believe that this morbid exhibition is but the effect of a peculiar septic poison existing in the atmosphere during epidemic visitation, that when taken into the system, act primarily upon the blood, producing a vitiated or semi-vitalized state of that fluid, by depriving it of its fibrin and leaving its serum in excess, so that it percolates, or is effused into the outer mucous membrane, through the minute capillaries and walls of the vessels, and remains upon the surface in a dissolved, semi-putrescible condition. In ordinary epidemic sorethroats the peculiar granular—yet superficial infiltration of the epithilium, has but little tendency to spread, and should not excite our fears—especially during an epidemic of scarlatina or diphtheritis. The ordinary phlegm, secreted and covering the tonsils, uvula, soft palate, and neighboring parts—does not consist of fibrillated coagulum, but rather of an amorphous deposit containing no trace of structural, or cellular element, but consist entirely of granular material among which may be occasionally seen, small transparent vesicles resembling minute blood corpuscles.—These upon the slightest pressure, disappear, leaving behind no trace of a fibrile texture. In the diphtheritis concretion, however recent, is never gelatinous, but possesses, when first formed, a creamy consistence giving to the part the appearance of having been coated with a yellowish white paint. This soon acquires elasticity and thickness, creating at once a striking resemblence to that seen upon the throat in scarlatina. Albuminous urine—so constantly present in scarlatina—was also observed in two ot the cases included in this report, but was not sufficiently marked to justify further notice just here.

Dengue has its rheumatism, and its exanthem, but without phlogosis; measles has its eruption, a highly vascular state of the lining membrane of the trachea and larynx, its thickned, softened and ulcerated condition, together with physical prostration—then may we not consider measels, diphtheritis and "eruption break bone," but modifications, or stages, rather than a variety of scarlatina?

*Treatment*—Being greatly confused in the outset as to the best mode of treatment—instituted none in particular—hence I experimented rather than administered with confidence. Most remedies derived from books were put in practice, yet a close study of the nature of the disease under consideration, lead me to discard most of them. I therefore chose such as would tend to arrest the de-vitalizing activity of this septic poison, and also to present, as far as possible, the separation of the serum of the blood from its fibrin, hence, I used chlorate of potassa, confiding in its well known refrigerant, diuretic and antiseptic properties. I usually administered it as a mouth wash, in those cases where there was considerable fœtor oris, and purulent discharge from the nose in the proportion of sixteen grains to four ounces of water. It seemed to lessen the putrescent oder, and at the same time absorb the coagula or lymph, deposited upon the tongue and fauces. In conjunction with this, I gave, internally, acetic acid, for its potent influence in promoting digestion as follows:

℞. Acidum aceticum, ℥ iss.
Syrupi Simp., ℥ iv.
Aqua dist., ℥ iv.
M. Dose tablespoonful every three hours.

It also acts as an astringent upon the serous membranes, prevents effusion, and alters materially, the plasticity of the blood.

*Saline Cathartics.*—These, so highly extolled by some practitioners, found no favor, as they seem to increase rather than retard the already dissolved state of the blood.

*Nitrate of Silver*, in stick or solution is regarded by most physicians as the remedy, *par-excellence*, yet two applications in my own hands, and its effects seen in many others, satisfied me that its use was productive of more mischief than good, and in no one instance was the slightest benefit, on the contrary, its tendency was to aggravate the disease, and hasten it to a fatal termination: dryness of the fauces, tongue and surrounding parts, invariably followed its use besides, increasing the already distressing dysphagia.

The end, said to be accomplished, was to arrest the spread of the deposit, and at the same time act as a direct absorbent, yet none of the supposed good effects were observed, or obtained. I therefore discarded it, and I am also convinced, that in every case where it is applied, death will inevitably ensue.

The only period in which I could be induced to apply so potent a remedy, is in the very outset of the disease when there is no engorgement of the lymphatics, and but slight red efflorescence. The existence of simple sore throat does not indicate its use. Should one venture its application, at all hazzards, because it is popular, and advised by many of the ablest physicians, I would suggest a very weak solution—say five or ten grains to the fluid ounce—and that applied in acute or inflammatory stage, and never after the membrane has been formed.

The known stimulant, vesicant, and escharotic effect of this salt, when applied to abraded surfaces, warts, strictures of the urethra, warty, excrescences fungus flesh, incipient chancres, and as a topical remedy in various external inflammations, but especially in erysipelas—applied both to the inflamed as well as surrounding healthy parts—rendered it popular in this, a disease purely of an epidemic nature, and dependent upon a peculiar atmospheric poison for its origin and importance—with the belief that its application alone to these surfaces would reach the seat of the disease, and thus arrest at once, its further progress. We must direct our attention to another channel, the system.—To effect this we should keep steadily in view, the primary indications favorable to alter and modify the already de-vitalized state of the blood, and prevent the further deposit of its morbid eliment. Should one fail in this, then hasten the absorption or discharge of the latter, by the administration of nutrition and medicinal stimulants, noting at the same time, with peculiar regard, the state of the digestion, as the system is slow to yield to medication, unless the stomach and bowels be fitted to discharge their chyliferous office. To this end, then, in the disease before us, we should act on them first, so as to prepare the economy for the reception of tonics, so called, such as preparations of cinchona, chlorate of potassa and the alkalies.

These, with many other remedies, have been tried, from the simplest to the most heroic, with occasional benefit, yet when the disease is suddenly aggravated, with abundant exudation from the nose, fetid breath, rapid pulse and great depression, all remedies will succumb, whatever you do. In such cases, the only reliance must be placed on a generous treatment, upon teas, broths, quinine, brandy, and especially upon a strengthening alimentation.

